# Quality of Life following Chemoradiotherapy for Localized Muscle Invasive Bladder Carcinoma: A Systematic Review

**DOI:** 10.3233/BLC-210011

**Published:** 2021-12-13

**Authors:** Ben-Max de Ruiter, Abel N. Keijzer, Maarten C.C.M. Hulshof, Adriaan D. Bins, Theo M. de Reijke, Jorg R. Oddens

**Affiliations:** a Department of Urology, Amsterdam University Medical Centers, University of Amsterdam, Amsterdam, The Netherlands; b University of Amsterdam, Amsterdam, The Netherlands; c Department of Radiation Oncology, Amsterdam University Medical Centers, University of Amsterdam, Amsterdam, The Netherlands; d Department of Medical Oncology, Amsterdam University Medical Centers, University of Amsterdam, Amsterdam, The Netherlands

**Keywords:** Urinary bladder neoplasms, quality of life, chemoradiotherapy, patient reported outcome measures

## Abstract

**BACKGROUND:**

Health Related Quality of Life (HRQoL) is an important factor regarding treatment for localized Muscle Invasive Bladder Carcinoma (MIBC), as it may affect choice of treatment. The impact of chemoradiotherapy (CRT) for MIBC on HRQoL has not yet been well-established.

**OBJECTIVE:**

To systematically evaluate evidence regarding HRQoL as assessed by validated questionnaires after definitive treatment with CRT for localized MIBC.

**METHODS:**

We performed a critical review of PubMed/MEDLINE, EMBASE, and the Cochrane Library in October 2020. Two reviewers independently screened articles for eligibility and assessed the methodological quality of the included articles using Joanna Briggs Institute critical appraisal tools. A narrative synthesis was undertaken.

**RESULTS:**

Of 579 articles identified, 11 studies were eligible for inclusion, including three RCTs and 8 non-randomized studies, reporting on HRQoL data for 606 CRT patients. Global health declined at End of Treatment (EoT), and recovered 3 months following treatment. Physical function declined from baseline at EoT and recovered between 3 and 24 months and was maintained at 5 years follow up. CRT had little effect on social and emotional function in the short-term, but HRQoL results in the long-term were lower compared to the general population. Urinary function declined from baseline at EoT, but returned to baseline at 6 months following CRT. After initial decline in bowel function, a complete return to baseline occurred 4 years following treatment. The majority of studies assessing sexual function showed no to little effect on sexual function.

**CONCLUSIONS:**

HRQoL recovers to baseline within 3 months to 2 years in almost all domains. The amount of available evidence regarding HRQoL following CRT for MIBC is limited and the quality of evidence is low.

## ABBREVIATIONS

BC: Bladder Cancer
BST: Bladder Sparing Treatment
CRT: Chemoradiotherapy
EoT: End of Treatment
Gy: Gray
JBI: Joanna Briggs Institute
MIBC: Muscle-invasive bladder cancer
PRISMA: Preferred Reporting Items for Systematic Reviews and Meta-analyses
PROs: Patient reported outcomes
PROMs: Patient reported outcome measures
HRQoL: Health related quality of life
RC: Radical cystectomy
RT: Radiotherapy
RoB: Risk of bias
TMT: Trimodal(ity) therapy
TURBT: Transurethral resection of a bladder tumor
WHO: World Health Organization

## INTRODUCTION

Bladder cancer is the ninth most frequent occurring cancer in the world [1]. Treatment guidelines still advocate radical cystectomy (RC) plus lymph node dissection as a primary treatment option for muscle invasive bladder cancer (MIBC) +/- neoadjuvant chemotherapy and indicate radiotherapy for frail patients [2]. Since the results of the BC2001 trial have been published in 2011, the focus is slowly shifting to chemoradiotherapy (CRT) as a serious alternative for radical cystectomy [3]. Currently, CRT, as a bladder sparing procedure, is offered as an alternative to selected, well-informed and compliant patients who are not willing to undergo a cystectomy and for whom radical cystectomy is not an safe option. In such a selected patient population, long-term survival rates of such a multimodality treatment (TURBT followed by CRT) are comparable to those of early cystectomy [4].

Besides the oncological outcomes of bladder cancer treatment, information about patient reported HRQoL is important when counselling patients, who are suitable candidates for both RC and CRT. Results of several validated questionnaires are available to assess various domains of HRQoL in patients who underwent RC for bladder cancer [5]. HRQoL outcomes following CRT for localized MIBC have not been studied as extensively as compared to RC in these patients, although in several smaller CRT studies HRQoL data have been reported. The primary objective of this review is to systematically evaluate all available evidence on HRQoL measured by validated cancer-specific questionnaires following CRT for localized MIBC.

## MATERIALS AND METHODS

### Search strategy

The review was performed according to the Preferred Reporting Items for Systematic Reviews and Meta-Analyses (PRISMA) statement [6]. A clinical librarian was involved whilst performing the search. Keywords used in the search were: *muscle invasive bladder cancer*, *chemoradiotherapy*, *trimodality, trimodal, bladder sparing* and *quality of life*. Synonyms and additional terms were used to broaden the search. Databases searched were EMBASE, MEDLINE and the Cochrane Library. The search was performed in May 2020 and updated in January 2021, without time-restrictions. Full details of the search strategies are available in the supplemental tables. All abstracts and full-text articles were screened using Endnote vX9.0 by two reviewers independently (B.R. and A.K.). Disagreement was resolved by discussion. If agreement could not be reached, a third independent party was consulted (J.O.).

### Eligibility criteria

Randomized and non-randomized studies were included. The following articles were included: (i) articles written in English, (ii) articles that included patients with T2-T4 non-metastatic muscle-invasive urothelial bladder carcinoma, (iii) articles that included adult (≥18 years of age) patients who were treated with radiotherapy in combination with chemotherapy and (iv) articles in which HRQoL was measured using a validated questionnaire. Reviews and conference abstracts were judged not to be eligible for inclusion. Studies that only used disease-specific HRQoL questionnaires that were not validated for bladder cancer patients were excluded. Generic questionnaires and pan-cancer questionnaires were included. Reasons for exclusion after full-text screening were recorded.

### Critical appraisal and risk of bias (RoB) assessment

The methodological quality of the included articles was assessed independently by two reviewers (B.R. and A.K.) using critical appraisal checklists from the Joanna Briggs Institute (JBI) [7]. Disagreement after the critical appraisal between the two independent reviewers was solved through discussion. If agreement could not be reached through discussion, a third independent assessor (J.O.) was consulted as arbiter. After completing the critical appraisal of the included studies, two reviewers (B.R. and A.K.) determined the number of questions of each checklist that had to be answered with *yes* for a study to have a high, moderate, or low RoB. Checklists were thoroughly reviewed and cut off values were determined based on discussion between reviewers. Additional potential biases were discussed and recorded.

### Data extraction and analysis

Data was collected independently by two authors (B.R. and A.K.) using a data-extraction form that was developed in accordance with the methodology described by the European Association of Urology [8]. Table 1 lists the data-points that were extracted. All studies were then narratively synthesized based on the following six HRQoL domains: global health, physical functioning, emotional functioning, social functioning, urinary function, bowel function and sexual function. If baseline data were described for included studies, HRQoL data were reported as decline or improvement from baseline. If statistical testing was performed, statistical significance was reported. If relevant data could be extracted from randomized studies, a meta-analysis was planned.

**Table 1:** Data points collected

Study characteristics	Author
	Year of publication
	Country
	Journal
	Study design
	Setting
	Enrolment period
Patient characteristics	Inclusion and exclusion criteria
	Number of patients
	Number of patients that received a HRQoL questionnaire
	Age
	Gender
	Tumor stage
	Follow-up period
Chemotherapy characteristics	Type
	Dosage
	Frequency
	Duration
Radiotherapy characteristics	Type
	Dosage
	Frequency
	Duration
HRQoL characteristics	HRQoL instruments
	Time points of HRQoL assessment
	HRQoL outcomes

## RESULTS

### Study selection

**Fig. 1:**
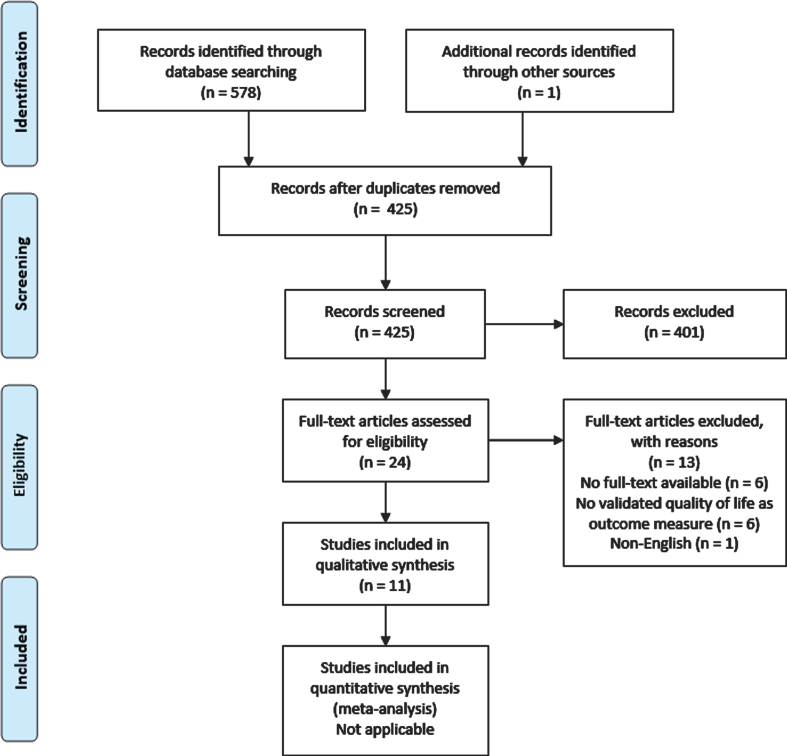
PRISMA flow diagram.

The study selection process and reasons for exclusion are outlined in Fig. 1. A total of 11 articles was included in the systematic review. In 6 cases the full-text articles could not be obtained after extensive search. If available, the corresponding email-addresses of the authors were contacted. In none of the cases extra data was obtained.

### Study characteristics

Table 2 presents the combined study and patient characteristics for the eleven articles that reported on HRQoL outcomes following CRT. We identified three RCTs and eight non-randomized studies. Due to the small number of articles and heterogeneity in the HRQoL data available, a meta-analysis could not be performed. Consequently, data was summarized narratively.

**Table 2:** Study Characteristics Abbreviations: RCT = randomized controlled trial; CS = cross sectional; P = prospective; RCS = retrospective case series; IQR = interquartile range; NR = not reported; CRT = chemoradiotherapy; MMC = mitomycin C; 5-FU = 5-fluorouracil; MC = multicenter; SC = single center

StudyID, country	Design	Setting	Follow-up (range)	CRT patients	Age (range)	T-stage %	Chemotherapy	Radiotherapy	Response rate (%)	CTCAE ≥3, %	Females %
Prospective studies											
Huddart (2020), (9) UK	RCT	MC	69.9 mo	182	72.9 (IQR 65.6–77.6)	T2: 85	MMC, 5-FU, concurrent	55 Gy / 64 Gy, continuous	1 year: 70	36	19
			(IQR 50.1–84.1)			T3: 12
						T4a: 4
Gogna (2018), (10) Aus	RCT	SC	48 mo (3 –87)	38	< 70 y: 19%	T2: 63	Cisplatin, concurrent	64 Gy, continuous	NR	NR	16
					70–79 y: 66%	T3: 29
					> 80: 16%	T4a: 8
Huddart (2017), (11)UK	RCT	MC	58 mo	20	63.2 (range 37.9 –75.2)	T2: 70	Gemcitabin, cisplatin, induction	55 / 64 Gy, continuous	NR	36	10
			(IQR 44.3 –61.3)			T3: 20
						Missing: 10
El-Sayed (2013), (15) Egy	P	SC	NR	36	55 (36–75)	T2: 72	Gemcitabin, concurrent	66 Gy, continuous	NR	NR	19
						T3: 28
Lagrange (2011), (14) Fra	P	MC	8 y	53	68 (43–78)	T2: 78	Cisplatin, fluorouracil, concurrent	45 Gy+18Gy boost	6	Grade 4: 8	11
						T3: 16
						T4a: 6
Herman (2004), (13) US	P	SC	NR	24	62 (46–83)	T2: 100	Gemcitabin, concurrent	60 –66 Gy, Continuous	92	22	0
Caffo (2003), (12) Italy	P	SC	19 mo (4 –43)	16	64 (55–75)	≥T2: 88	Cisplatin, gemcitabin, concurrent	54 Gy, continuous	NR	81	13
						T4: 13
Retrospective studies											
Mak (2016),(16) US	CS	MC	5.6 y	74	76 (IQR 69–81)	T2: 94	NR	64 Gy (median), alternating regimens	86	NR	22
						T3: 6
Hashine (2008),(17) Jap	CS	SC	47.4 mo (6.1–152.6)	48	70 (46–84)	NR	Cisplatin, pirarubicin	44 Gy, split course	69	NR	15
Zietman (2003), (18) US	RCS	SC	6.3 y (1.6 –14.9)	71	68.9 (70.9–77.5)	T2: 68	Cisplatin, 5-FU, MCV	64–65 Gy, alternating regimens	68	NR	26
						≥T3: 32
Caffo (1996), (19) Italy	RCS	SC	31mo (6 –68)	15 CRT (44 RT)	72 (40–86)	NR	MCV	60–65 Gy, continuous	66	NR	21

### Critical appraisal and risk of bias (RoB) assessment of the included studies

**Fig. 2:**
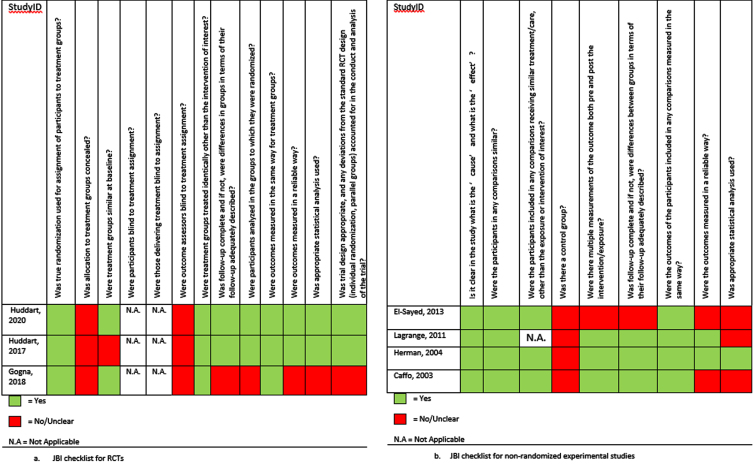
Critical appraisal of prospective studies using the JBI checklists.

**Fig. 3:**
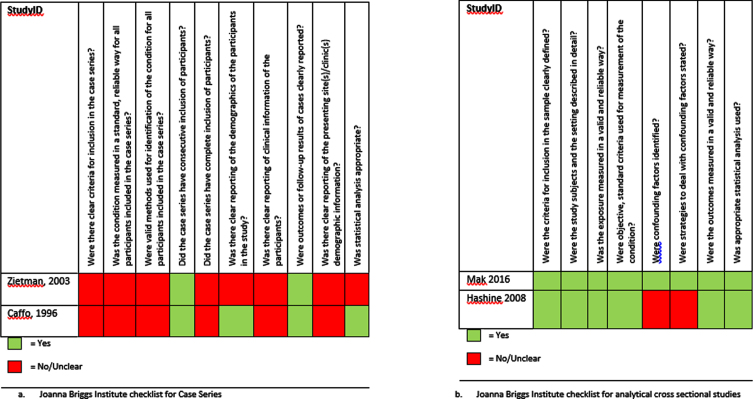
Critical appraisal of the retrospective studies using the JBI checklists.

Figures 2 and 3 display the critical appraisal per checklist that was used.

Figure 2a displays the critical appraisal for the three RCTs. As it was not possible to blind participants to their intervention, all RCTs had a high RoB for blinding. The reviewers agreed that the lack of blinding did not influence the study quality for all RCTs. The study by Huddart et al. (2020) was deemed to have a low RoB [9]. Two of the RCTs did not complete recruitment. Of those, Gogna et al. (2018) reported results of their trial in a letter to the editor, not fully describing the methodology of the trial. Therefore, the study was deemed to have a high RoB [10]. The SPARE trial also did not fulfil complete recruitment, and due to a high number of changes in treatment allocation following randomization the study was deemed a moderate RoB [11].

The critical appraisal of the prospective non-randomized studies is displayed in Fig. 2b. Due to the nature of the research, all non-randomized phase 1–2 studies were at high risk of selection and performance bias [12–14]. The study by Herman et al. (2004) had a low RoB [13], the study by Lagrange et al. (2012) and Caffo et al. (2003) were deemed a moderate RoB [12, 14]. Finally, the study by El-Sayed et al. (2013) had a high RoB [15].

The critical appraisal of the retrospective studies is shown in Fig. 3. All retrospective studies were deemed a high risk of selection, attrition and performance bias. Of those studies, Mak et al (2016) had a low RoB, Hashine et al. (2008) had a moderate RoB, and Zietman et al. (2003) and Caffo et al. (1996) were scored a high RoB [16–19].

### Types of HRQoL questionnaires

The following validated HRQoL instruments were reported by the studies included in this systematic review: the EORTC QLQ-C30 [20] was used by six studies [10–12, 14, 16, 19], the EORTC QLQ-BLM30 [21] by three studies [10, 11, 16], the SF-36 [22, 23] by two studies [17, 18] and the FACT-G [24] by one study [13]. The FACT-BL [25] was used by two studies [9, 13] and the NCCN-FACT FBISI18 [26] by one study [15]. Caffo et al. (1996) [19] used a HRQoL questionnaire based on the EORTC QLQ-C30 questionnaire and added items of other questionnaires to the newly-constructed HRQoL questionnaire. The questionnaire was validated in their study and, therefore, it was included in this systematic review [12]. Supplemental Table 1 provides an overview of the questionnaires used, the HRQoL domains that were extracted, and the time of questionnaire administration per study.

### Results of HRQoL measurements

Two studies provided mean HRQoL scores following CRT, without comparing those scores to baseline HRQoL scores or HRQoL scores of the overall population. Mak et al. presented mean HRQoL scores with a mean time of 9 years following CRT and El-Sayed et al. assessed HRQoL in MIBC patients 3 months following CRT [15, 16]. The relevant HRQoL scores of both studies are presented in supplemental Tables 2 and 3.

**Fig. 4a-g:**
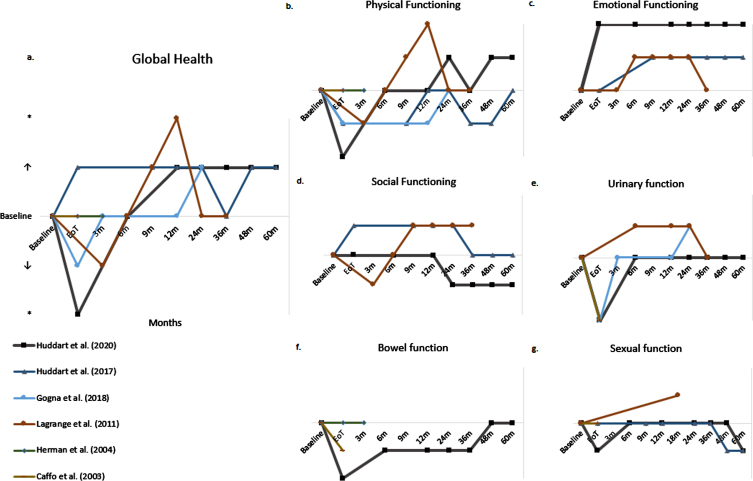
Change of baseline over time per HRQoL domain of prospective studies; ↑: indicates trend towards improved HRQoL; ↓: indicates trend towards declined HRQoL”; ^*^ indicates significant improvement or decline, m: month.

Patient reported HRQoL questionnaires measure/assess HRQoL outcomes in different domains: *global health, physical function, emotional function, social function, urinary function, bowel function* and *sexual function.* Results per domain were collected. Figure 4 displays the change in HRQoL from baseline over time per HRQoL domain in the prospective studies.

### Global health

Six studies reported data on global health [10, 11, 14, 16–18]. Figure 4a displays the outcome over time of the prospective studies. One of two RCTs reported an initial decline in global health score at the end of treatment period, which returned to baseline 3 months following CRT and was maintained at long-term follow-up [10]. The other RCT reported slightly better global health scores at the end of treatment, compared to baseline [11]. Both RCTs showed that global health scores were maintained above baseline at long-term follow up of 2 to 5 years and indicated an improvement of global health compared to baseline.

One prospective study also reported an initial decline in global health score, which recovered to baseline 6 months following CRT. At follow up to 3 years, global health scores were maintained above baseline and indicated an improvement of global health compared to baseline [14].

Two studies showed no significant improvement or decline in global health scores 47.4 months and 6.3 years following CRT compared to the age-matched overall Japanese and American population, respectively [17, 18].

### Physical functioning

Ten studies assessed physical functioning [9–12, 14–19]. Figure 4b displays physical functioning over time assessed by the prospective studies. Two out of three RCTs reported an immediate significant decline of physical functioning following CRT. Two of three RCTs reported a recovery to baseline at 6 and 24 months following CRT, respectively [9, 10]. Huddart et al. (2017) reported similar data, with a recovery to baseline at 12 months following treatment, followed by a decline at 36 months to return to baseline only at 5 years after treatment [11].

Lagrange et al. (2011) reported similar data with an initial decline followed by recovery to baseline at 6 months, which was maintained up to 36 months [14]. Furthermore, Caffo et al. (2003) did not find a statistically significant difference between physical functioning before CRT and 19 months (median) following CRT [12].

Two studies showed that 47.4 months and 6.3 years following CRT physical functioning was not significantly worse than in an age-matched Japanese and American cohort, respectively [17, 18].

Finally, Caffo et al. (1996) showed that 2.6 years after CRT, 69% and 62% of patients felt much to very much energetic and physically well, respectively. And 3% of patients had much to very much physical pain, 10% of patients felt much to very much ill and 10% of patients felt a little to very tired [19].

### Emotional functioning

Six studies reported on emotional functioning [9, 11, 14–17]. Figure 4c displays emotional functioning over time assessed by the prospective studies. Three prospective studies showed that emotional functioning improved following CRT. Two RCTs reported that emotional functioning remained stable at long-term follow-up of 5 years [9, 11]. Lagrange et al. (2011) reported that emotional functioning declined 2.5 years following CRT [14].

Long-term cross-sectional results showed that after a median of 47.4 months following CRT, emotional functioning was statistically significant worse in patients between 70 and 80 years compared to the age-matched Japanese population [17].

### Social functioning

Five studies reported on social functioning [9, 11, 14, 16, 17]. Two RCTs showed no statistically significant change from baseline at multiple time points up to 5 years following CRT [9, 11].

A prospective study by Lagrange et al. showed that after an initial slight decline following CRT, social functioning increased 3 months later and remained stable 3 year follow-up. All changes reported from baseline were not statistically significant [14].

One cross-sectional study found that 47.4 months following CRT, patients between 70 and 80 years had significantly worse social functioning compared to the age-matched Japanese population [17].

### Urinary function

Nine studies assessed the HRQoL domain “urinary function” [9, 10, 12–16, 18, 19]. Two RCTs reported significant worsening of urinary symptoms at the end of treatment. Recovery to baseline occurred quickly. At 6 months no significant change was observed anymore, and this was maintained at 3–5 years of follow-up [9, 10].

One of the three prospective non-randomized studies confirmed this significant decline in urinary function at the end of treatment [12]. Lagrange et al. (2011) reported an improvement of symptoms from 6 months following treatment onwards, which was maintained at 3 years follow up [14]. Herman et al. (2004) did not report a significant decline. Their group did show that 43% of patients experienced an increase in urinary urgency during treatment and 85% of patients experienced an increase in urinary frequency during treatment. At the end of treatment, 50% of patients reported quite a bit or more urinary frequency, compared to 21% at baseline [13].

A retrospective study by Caffo et al. (1996) reported that 2.6 years following CRT 3% and 0% of patients experienced much to very much dysuria and hematuria, respectively [19]. Forty-four percent of patients experienced a high to very high daily frequency, 42% experienced a much to very much nightly urination and 38% reported a much to very much urinary control problems.

Zietman et al. (2003) reported that 6.3 years following CRT urinary symptoms reflected gender differences also found in the general population. One-fifth of patients reported some degree of incontinence within the preceding 7 days. Incontinence was two times more frequent in women [18].

### Bowel function

Four studies assessed the HRQoL domain “bowel function” [9, 12, 13, 18]. One RCT reported that 19% of patients did not have control over their bowel function (baseline 12%) and that 12% of patients had diarrhea (baseline 1.2%) at the end of treatment. At 1 year follow-up, 14% of patients did not have control over their bowel function and 2.1% of patients had diarrhea [9].

Caffo et al. (2003) reported lower scores on the rectal subscale at the end of treatment [12]. The other prospective study reporting on bowel function found that bowel function was unchanged from baseline in 71% of patients at 3 months following treatment [13]. One retrospective study reported that *difficult control* (22% of participants) was most frequent 6.3 years following CRT [18].

### Sexual function

Seven studies reported on sexual function [9, 11–14, 16, 19]. Two RCT’s reported no significant negative effects from CRT on sexual function [9, 11]. One RCT reported that 1 year following CRT, 21% of males were able to have or maintain an erection (baseline 24%). At 5 year follow-up this percentage had decreased to 10%. No statistical testing was performed.

Caffo et al. (2003) suggested that treatment with CRT did not affect sexual function [12]. Lagrange et al. found that 11 of 14 (79%) respondents had a preserved sexual function 18 months following CRT against 24 of 43 (56%) of patients at baseline [14]. Herman et al. (2004) reported that 58% of patients had an unchanged erectile function at end of treatment compared to baseline [13]. One retrospective study reported that 2.6 years following CRT, 26% of patients experienced much to very much reduction in sexual activity and 25% of men experienced a much to very much erection problems [19].

## DISCUSSION

The aim of this systematic review was to synthesize and provide an overview of the available evidence on HRQoL following CRT for MIBC. This systematic review examined 11 studies assessing HRQoL following CRT with validated cancer-specific HRQoL questionnaires. We compared different HRQoL questionnaires based on deviation from baseline, or comparison to other studies that applied the same health domains.

Overall, global health was maintained above baseline at long-term follow up of 5 years. Studies that showed an initial decline following CRT found that recovery to baseline occurred 3 to 6 months following CRT. Physical functioning declined immediately following CRT, but a majority of studies showed that recovery to, or above, baseline occurred between 6 months to 2 years following CRT and that physical functioning remained stable at long-term follow-up until 5 years following treatment [9, 10, 14]. Huddart et al. (2017) suggested that 36 months following CRT physical functioning declined, recovering to baseline only 5 years following treatment. This observation is based on data of only 16 patients that was available for follow-up. Results may, therefore, be skewed due to undersampling. At 5 years follow-up only 50% of patients were alive, which may also induce survivor bias [11]. However, various included studies that measured physical functioning in the long term substantiate the finding of good physical functioning following CRT in the long run [12, 17–19].

Emotional functioning improved following CRT and seemed to stay above baseline at long-term follow-up [9, 11, 14]. However, Hashine et al. found that emotional functioning was significantly worse until 47.4 months (median) following CRT in patients between 70 and 80 years compared to the age-matched Japanese population [17]. These conflicting results could indicate the presence of survival bias in prospective studies reporting on MIBC patients that have been treated with CRT. Social functioning seemed to improve following CRT, but results of long-term social functioning are conflicting [9, 11, 14].

When regarding social function as a domain of HRQoL, Hashine et al. showed that this was significantly worse in patients between 70 and 80 years until 47.4 months (median) following CRT compared to the age-matched Japanese population [17]. A large study into the HRQoL of bladder cancer patients also suggested worse social functioning in MIBC patients that underwent radiotherapy [27]. A possible explanation may be the urinary symptoms and bowel symptoms that can be induced by CRT treatment, which might explain the hindrance to social functioning.

Three of the described studies reported a significant proportion of patients that suffered from urinary symptoms following CRT. Urgency, frequency and incontinence were most prevalent [12, 13, 19]. However, urinary function appears to improve at longer follow-up following CRT and remains stable thereafter [9, 10]. Studies that evaluated bowel function reported lower scores following treatment. However, these findings were not statistically significant.

Impaired bowel control was present in more than one fifth of patients 6.3 years following CRT [11–13]. Noteworthy is that long-term results may not adequately reflect toxicity profiles that are to be expected of current, improved radiotherapy regimens. Recent advantages in radiotherapy techniques, such as intensity modulated radiotherapy (IMRT) and the use of a partial bladder boost, reduce radiation on surrounding tissue, thereby possibly minimizing radiation induced toxicity and thus potentially improving HRQoL [28].

A majority of studies did not reveal a decrease in sexual functioning at 1-1,5 year follow up. However, the largest RCT reported that the ability to maintain an erection decreased in the 5 years following CRT from 21% to 10% [9] and long-term results of some non-randomized studies suggested a decrease in sexual activity and difficulty in maintaining an erection in a quarter of patients [19]. These long-term results may at least partly reflect the natural course of ageing in the elderly, often comorbid population, and thus is unlikely to be attributed solely to the treatment [10, 11]. Lagrange et al. (2011) even reported a 23% higher number of patients with adequate sexual function 18 months following CRT then before CRT. A possible explanation may be selection bias due to the fact that only 14 patients completed the single question on sexual activity at 18 months, compared to 43 respondents before treatment [14]. Other prospective non-randomized studies supported the outcomes found in the randomized studies [12, 13]. Interestingly, none of the studies reported data on female sexuality. A recent paper by Catto et al. (2021) on HRQoL in BC patients showed that females are reluctant to answer these questions, thereby confirming that the effect of treatment on sexual HRQoL in women is poorly understood [29].

A comparison of HRQoL following CRT for MIBC with patients who underwent a RC proves to be difficult, as these data are limited. Thus far, only one study, included in this review, compared HRQoL following RC with selective bladder preservation(SBP). The authors suggested that patients who received radiotherapy showed improvement in global health and social functioning 12 months following treatment, whilst these declined following RC. Changes over time furthermore suggested a decline in body image and male sexual problems that is less evident for the patients receiving RT. However, patient numbers in both groups were low and confidence intervals overlapped, thereby not allowing any conclusions [11]. Interestingly, the largest systematic review reporting on HRQoL following RC, including data on 3754 patients, reports that overall HRQoL improves over the first 12 months following RC, after an initial immediate decline. The HRQoL starts to diminish 12 months postoperatively in all domains [30]. We did not find a similar trend following CRT for MIBC. Although in most studies a recovery of HRQoL to baseline is observed within 6 months to 2 years for all domains, except bowel function, these results should be interpreted with caution. We found that there was a significant heterogeneity in HRQoL questionnaires used in the included studies, as well as the time point of administering the questionnaires and the numbers of questionnaires completed. Furthermore, statistical significance in HRQoL research does not automatically translate into clinically important difference [31, 32]. Only one article performed an exploratory analysis into a minimally clinically significant change from baseline, to determine a relevant change in HRQoL scores. The authors based their definition of clinically significant change on previous work on FACT questionnaires [9]. Furthermore, HRQoL research is known to have several biases, such as acquiescence (respondents tend to agree to items) and extreme response style (respondents tend to give extreme responses). In addition, prospective HRQoL research is susceptible to bias such as response shift, or recalibration response, where participants adapt to their current situation. The use of retrospectively gathered questionnaires is known to have recall bias, where participants remember their former state better or worse than it actually is [33].

We found considerable heterogeneity in CRT schedules which can also influence HRQoL outcomes. The studies included in this review adhered to different chemotherapy regimens, which influence toxicity and, therefore, might influence patient reported HRQoL.

The overall quality of evidence is low in the available literature. Two out of 3 RCTs were prematurely stopped due to failed recruitment. Prospective studies are mostly limited to phase 1–2 studies that include WHO performance status 0–1 patients that might not represent the overall MIBC population. Retrospective research has well-known limitations, of which selection bias is most prevalent. With an increased focus on bladder-sparing alternatives for MIBC and HRQoL, it is to be expected that more high-quality HRQoL research is to follow in the coming years.

The strengths of this systematic review are the extensive search in three databases without time restriction, limiting the opportunity of missing relevant publications. Furthermore, the review was conducted according to a systematic approach, adhering to the PRISMA guidelines [6]. Finally, this review focuses specifically on patient reported HRQoL measures, excluding physician related bias in outcome results.

This systematic review has, however, several limitations that should be mentioned. Firstly, the amount of CRT patients was low in most of the included studies. Only four studies reported HRQoL outcomes of more than 50 patients [9, 14, 16, 18]. Furthermore, most studies adhered to different chemotherapy and radiotherapy regimens, which hampers comparison. Secondly, although deliberately, only articles written in English were included and conference abstracts were not included in this systematic review. During screening on title and abstract, we found two conference abstracts that reported HRQoL following CRT, but no published full-text articles were available [34, 35]. This could indicate publication bias. Furthermore, there is severe methodological heterogeneity amongst studies. Finally, in contrast to male sexual function, no specific data on female sexual function was reported in any of the studies, thereby making it impossible to draw conclusions in this domain.

To our knowledge, this is the first systematic review presenting the impact of CRT for non-metastatic localized MIBC on health-related HRQoL measured by validated cancer-specific questionnaires only. Feuerstein et al. (2015) published a review on HRQoL following radiotherapy-only for MIBC, but included non-validated questionnaires. In that report, no randomized studies were available [36].

Although this systematic review suggests that overall HRQoL following CRT for MIBC is maintained, high quality evidence supporting this suggestion is limited thus far. The authors propose performing exploratory analysis into minimally important differences before reporting on HRQoL research for MIBC. A Core Outcome Set, which has been published for several specific cancer fields, such as for prostate cancer efficacy trials, can minimise confounding due to inconsistencies in the selection, definition, measurement and reporting of outcomes in clinical trials [37, 38]. Development of a Core Outcome Set for muscle invasive bladder cancer trials can contribute to better outcome reporting for HRQoL following treatment using CRT for MIBC.

In summary, our recommendations for future studies into HRQoL following MIBC treatment would include adequate sampling in general, accounting for confounding variables. In addition, comparing arms with RC, the general population, and new future combinations, such as with checkpoint inhibition are needed. Finally, consistent study methodology with baseline values, with exploratory analyses into clinically relevant change from baseline would improve outcome reporting and thereby help in counselling MIBC patients in their choice of treatment.

## CONCLUSIONS

This systematic review shows that, in general, HRQoL following CRT recovers to baseline or above, within 6 months to 2 years in almost all domains. However, the review also shows that the amount of available evidence addressing HRQoL following CRT for MIBC is limited. In addition, the overall quality of the available evidence was low and results were difficult to synthesize due to different treatment regimens. Regarding the current data on CRT outcomes, more prospective phase 3 trials are needed to establish robust conclusions on HRQoL following CRT for MIBC, and include a comparative arm with radical cystectomy.
